# eIF2α-CHOP-BCl-2/JNK and IRE1α-XBP1/JNK signaling promote apoptosis and inflammation and support the proliferation of Newcastle disease virus

**DOI:** 10.1038/s41419-019-2128-6

**Published:** 2019-11-26

**Authors:** Yanrong Li, Weiyu Jiang, Qiaona Niu, Yingjie Sun, Chunchun Meng, Lei Tan, Cuiping Song, Xusheng Qiu, Ying Liao, Chan Ding

**Affiliations:** 1 Department of Avian Diseases, Shanghai Veterinary Research Institute, Chinese Academy of Agricultural Sciences, Shanghai, 200241 P. R. China; 2 Jiangsu Co-innovation Center for Prevention and Control of Important Animal Infectious Diseases and Zoonoses, Yangzhou, 225009 P. R. China

**Keywords:** Infection, Infection

## Abstract

Newcastle disease virus (NDV) causes severe infectious disease in poultry and selectively kills tumor cells, by inducing apoptosis and cytokines secretion. In this report, we study the mechanisms underlying NDV-induced apoptosis by investigating the unfolded protein response (UPR). We found that NDV infection activated all three branches of the UPR signaling (PERK-eIF2α, ATF6, and IRE1α) and triggered apoptosis, in avian cells (DF-1 and CEF) and in various human cancer cell types (HeLa, Cal27, HN13, A549, H1299, Huh7, and HepG2). Interestingly, the suppression of either apoptosis or UPR led to impaired NDV proliferation. Meanwhile, the inhibition of UPR by 4-PBA protected cells from NDV-induced apoptosis. Further study revealed that activation of PERK-eIF2α induced the expression of transcription factor CHOP, which subsequently promoted apoptosis by downregulating BCL-2/MCL-1, promoting JNK signaling and suppressing AKT signaling. In parallel, IRE1α mediated the splicing of XBP1 mRNA and resulted in the translation and nuclear translocation of XBP1s, thereby promoting the transcription of ER chaperones and components of ER-associated degradation (ERAD). Furthermore, IRE1α promoted apoptosis and cytokines secretion via the activation of JNK signaling. Knock down and overexpression studies showed that CHOP, IRE1α, XBP1, and JNK supported efficient virus proliferation. Our study demonstrates that the induction of eIF2α-CHOP-BCL-2/JNK and IRE1α-XBP1/JNK signaling cascades promote apoptosis and cytokines secretion, and these signaling cascades support NDV proliferation.

## Introduction

The endoplasmic reticulum (ER) plays an important role in regulating protein synthesis/processing, lipid synthesis, and calcium homeostasis^1^. During virus infection, many viral proteins are synthesized by ER-associated ribosome and transported into the ER lumen for proper folding or posttranslational modification. This leads to an overwhelming load of unfolded or misfolded proteins in the ER lumen. The chaperone Bip then binds to these unfolded/misfolded proteins and releases the ER stress sensors PERK, ATF6, and IRE1α, triggering unfolded protein response (UPR). PERK is activated by autophosphorylation and in turn phosphorylates eIF2α on Ser51^2,3^. Phospho-eIF2α has increased affinity to the eIF2β subunit and prevents the regeneration of guanosine triphosphate (GTP) in the ternary complex eIF2-GTP-^Met^tRNAi, thus halting the initiation of protein translation^4^. However, ATF4 mRNA is preferentially translated and promotes the transcription of genes important for cellular remediation or apoptosis, including CHOP^5^. Another eIF2α kinase, PKR, also elicits eIF2α-ATF4-CHOP signaling in response to virus infection^6–8^. ATF6 dissociates from Bip and moves to the Golgi apparatus, where it is cleaved into N-terminal fragment ATF6-N and subsequently translocated to the nucleus as an active transcription factor, triggering the transcription of protein chaperones and components of ER-associated degradation (ERAD)^9–11^. The IRE1α-XBP1 branch is the most evolutionarily conserved in Eukarya^12^. After dissociating from Bip, IRE1α undergoes homo-oligomerization and autophosphorylation, obtaining both kinase and endoribonuclease activity^13^. This endoribonuclease leads to unconventional enzymatic splicing of XBP1u mRNA into XBP1s by removing the 26-nucleotide intron. The spliced mRNA is then translated into transcription factor XBP1s, controlling the expression of the ER quality control genes and components of ERAD^11,13–15^. IRE1α also degrades ER-associated mRNAs to attenuate protein load in the ER lumen^16^.

If ER homeostasis cannot be restored, UPR drives the damaged or infected cells to apoptosis^17^. Apoptosis is triggered by either intrinsic signaling or extrinsic death ligands. The intrinsic pathway is under the control of the BCL-2 protein family^18^. Pro-survival guardian proteins (BCL-2, MCL-1, and BCL-xL) inhibit apoptosis through binding and sequestering pro-apoptotic activators (BID, BIM, PUMA, and NOXA) or effectors (BAX and BAK). When enough activators have been stimulated by cytotoxic stresses, BAX is released from pro-survival guardian proteins and oligomerizes to form pores on the mitochondrial outer membrane, thereby releasing cytochrome c and activating caspase 9^19–21^. Under persistent ER stress, the induction of CHOP may promote cell apoptosis by regulating the expression of BCL-2, TRB3, death receptor 5, ERO1α, and GADD34, and perturbing the cellular redox state^22–24^. IRE1α may promote apoptosis through interacting with TRAF2 and ASK1, in turn activating pro-apoptotic JNK^25^. Prolonged UPR is also an inflammatory nidus and may elicit a defensive innate immune response against the invading pathogen. Activation of IRE1α-JNK leads to the increased expression of pro-inflammatory cytokines^26^. NF-кB is activated by IRE1α-TRAF2 mediated IκB phosphorylation and degradation^27^. In another way, when protein translation is halted by PERK-eIF2α signaling, the ratio of NF-кB to IкB is increased, and NF-кB is then released and activated^28,29^.

Newcastle disease virus (NDV) is a highly contagious avian pathogen belonging to the genus *Avulavirus* within the family *Paramyxoviridae*^30^. NDV infection caused death of chicken embryos and neurological damage in adult chicken are the consequences of apoptosis and inflammation^31^. NDV also selectively infects the human cancer tissues, kills cancer cells directly or attracts immune cells to remove the infected tumor cells^32^. Although several reports show that NDV infection results in the loss of mitochondrial membrane potential^33–36^ and the induction of extrinsic death ligands TNF-α/TRAIL^37^, the intrinsic death signals are yet to be fully clarified. We have reported that NDV infection-induced phosphorylation of eIF2α results in shut off of protein translation^38^. In this study, we focused on characterization of the UPR branches and their roles in NDV- triggered apoptosis and inflammation, in several human cancer cell types and avian cells.

## Materials and methods

### Cells and virus

The human cervical cancer cell line (HeLa), human non-small cell lung cancer cell lines (A549 and H1299), chicken embryo fibroblast monolayer cell line (DF-1), and human embryonic kidney cell line (293T) were purchased from ATCC (Manassas, VA, USA). Tongue squamous carcinoma cell line (CAL27), squamous cell carcinoma of oral cancer cell line (HN13), and human hepatocellular carcinoma cell lines (Huh7 and HepG2) were provided by Prof. Lijun Jia (Shanghai University of Chinese Medicine, shanghai, China). These cells were cultured in Dulbecco’s modified Eagle’s medium (DMEM) (Hyclone, USA), RPMI 1640, or F-12K medium supplemented with 10% fetal bovine serum (FBS, Gbico, USA) at 37 °C humidified atmosphere containing 5% CO_2_. Chicken embryo fibroblasts (CEFs) were derived from 10-days-old SPF chicken embryos which bought in MERIAL (France).

The NDV velogenic strain Herts/33 was obtained from China Institute of Veterinary Drug Control (Beijing, China). The virus was propagated in chicken embryonated eggs and titrated on DF-1 cells by TCID_50_ assay. The virus was used for infection at the multiplicity of infection (MOI) of 1 throughout this study.

### Reagents and antibodies

The IRE1α inhibitor 8-formyl-7-hydroxy-4-methylcoumarin (4µ8c) (s7272), PKR/PERK inhibitor GSK2606414 (s7307), JNK inhibitor SP600125 (s1460), ERK1/2 inhibitor U0126 (s1102), p38 inhibitor SB203580 (s1076) and AKT inhibitor LY294002 (s1105) were purchased from Selleck Chemicals (USA). 4-phenylbutyric acid (4-PBA) (B26966) was purchased from YuanYe Biological Company (China). RNA extraction reagent TRIzol®, transfection reagent Lipofectamine 2000, Click-iTTM Plus TUNEL Apoptosis Assay Kit (C10617), and Dead Cell Apoptosis Kit with Annexin V Alexa Fluor™ 488 & Propidium Iodide (PI) (V13245) were purchased from Invitrogen Thermo Fisher Scientific (USA). Western blot stripping buffer (p0025) and 4ʹ,6ʹ-diamidino-2-phenylindole (DAPI) (c1002) were purchased from Beyotime Biotechnology (China). SYBR Green qPCR Mix (p2092) was purchased from Dongsheng Biotech (China).

Monoclonal NDV NP antibody was raised in mice using bacterially expressed His-tagged NP as the immunogen. Antibodies against Caspase-3 (9665), PARP (9542), PERK (5683), phospho-eIF2α (3398), eIF2α (5324), IRE1α (3294), ATF6 (65880, used for western blot), CHOP (2895), PKR (12297), BCL-2 (4223), MCL-1 (5453), BCL-xL (2764), BIM (2933), PUMA (12450), BAX (5023), phospho-AKT (13038), AKT (4691), phospho-JNK (4668), JNK (9252), phospho-p38 (4511), p38 (8690), phospho-ERK1/2 (4370), ERK1/2 (4695), TBK1(3504), phospho-TBK1 (5483), IRF3 (11904), p65 (8242), and phospho-p65 (3033) were purchased from Cell Signaling Technology (USA). Phospho-PERK (DF7576) was purchased from Affinity Biosciences. Phospho-IRE1α (ab48187), XBP1 (ab37152), phospho-PKR (ab32506), phospho-IRF3 (ab76493), and ATF6 (ab122897, used for immunofluorescence) were purchased from Abcam (UK). Anti-Flag and β-actin (A1978) were purchased from Sigma-Aldrich (USA). The secondary IgG conjugated with HRP, FITC, or TRITC were obtained from DAKO (Denmark).

The specific sequences of small interfering RNA (siRNA) oligos of caspase-3, PERK, PKR, CHOP, AKT, JNK, p38, ERK1/2, IRE1α, XBP1, and non-target control siRNA (sic) were shown in Table 1. All siRNAs were synthesized by Gene Pharma Co. Ltd (Shanghai, China).

**Table 1:** Small interfering RNA (siRNA) sequence.

Name	Sequence (5ʹ–3ʹ)
sic	UUCUCCGAACGUGUCACGUTT
siPERK	GGUUGGAGACUUUGGGUUAUU
siPKR	GCGAGAAACUAGACAAAGUUU
siCHOP	GAGCUCUGAUUGACCGAAUTT
siIRE1α	CUCCGAGCCAUGAGAAAUATT
siXBP1	GGAACAGCAAGUGGUAGAUTT
siCASP3	GCAUAUCAGUUGAGCUUCATT
siAKT	GAAGGAAGUCAUCGUGGCCUU
siERK1	UGA CCA CAU CUG CUA CUU C
siERK2	GUG CUG UGU CUU CAA GAG C
sip38	GAACUGCGGUUACUUAAAC
siJNK	AAAGAAUGUCCUACCUUCUUU

*sic* Non-target control siRNA, *siCASP3* siCaspase 3

### Tissue culture infectious dose 50 (TCID_50_) assay

Virus yield in culture medium of NDV-infected cells was determined by measuring TCID_50_ in DF-1 cells. In brief, DF-1 cells were seeded in 96-well plates at a density of 2.0 × 10^4^ cells per well. After 24 h, cells were infected with virus, which was serially diluted in 10-fold using serum free medium. The virus and cells were incubated at 37 °C for 4 days. The cytopathic effect of cells was observed using light microscopy. TCID_50_ was calculated by the Reed-Muench method.

### TUNEL assay

The TUNEL method was performed to label the 3ʹ-end of fragmented DNA of the apoptotic cells. Different cell lines were infected by NDV Herts/33 at an MOI = 1 and harvested at 20 h post-infection (h.p.i.), respectively. The TUNEL assay was carried out by Click-iTTM Plus TUNEL Apoptosis Assay Kit according to the manufacture’s instruction. The images of TUNEL positive cells were captured by a fluorescence microscope (×200).

### Flow cytometry

Various cell lines were infected with NDV Herts/33 strain at MOI = 1, and harvested at 20 h.p.i. According to the manufacturer’s instruction, cells were stained with Annexin V and Propidium Iodide (PI) by using a Dead Cell Apoptosis Kit with Annexin V Alexa Fluor™ 488 & PI and analyzed with flow cytometry by using flow cytometer (Beckman) equipped with FlowJo software.

### Construction of plasmids

For construction of PXJ40F-CHOP plasmid, full-length CHOP (NM_004083.5) was amplified by PCR from human cDNA using forward primer 5′-CCCAAGCTTATGGCAGCTGAGTCATTGCCTTTC-3′ and reverse primer 5′-GGAAGATCTTCATGCTTGGTGCAGATTCACCATTC-3′. The restriction enzyme sites were underlined. The PCR product was digested with *Hind III* and *Bgl II*, ligated into vector PXJ40F (with a Flag tag in amino terminus). For construction of pCMV-IRE1α plasmid, full-length IRE1α (GenBank: AF059198.1) was amplified by PCR from human cDNA using forward primer 5′-GCAATCAAGCTTATGCCGGCCCGGCGGCTGCTGC-3′ and reverse primer 5′-GACGTGGAATTCGAGGGCGTCTGGAGTCACTGGGGGC-3′. The PCR product was digested with *Hind III* and *EcoR I*, ligated into vector p3ψFlag-CMV-14. For construction of pCMV-XBP1u plasmid, full-length XBP1u (NM_005080.3) was amplified by PCR from human cDNA using XBP1 forward primer 5′-GCAATCAAGCTTATGGTGGTGGTGGCAGCCG-3′ and XBP1u reverse primer 5′-GACGTGTCTAGAGTTCATTAATGGCTTCCAGCTTGGC-3′. The PCR product was digested with *Hind III* and *Xba I*, ligated into vector p3ψFlag-CMV-14. For construction of pCMV-XBP1s plasmid, full-length XBP1s (NM_001079539.1) was amplified by PCR from human cDNA using the forward primer 5′-GCAATCAAGCTTATGGTGGTGGTGGCAGCCG-3′ and reverse primer 5′-GACGTGTCTAGAGACACTAATCAGCTGGGGAAAGAG-3′. The PCR product was digested with the restriction enzyme *Pst I* to remove the XBP1u fragment, followed by *Hind III* and *Xba I* digestion, finally cloned into vector p3ψFlag-CMV-14.

### Transfection of plasmid or siRNA

HeLa cells were transfected with plasmids or siRNAs using lipofectamine 2000 reagent (Invitrogen, USA) according to the manufacture’s manual. At 24 h (plasmid transfection) or 36 h (siRNA transfection) post-transfection, cells were incubated with NDV in serum-free medium at 37 °C for 1 h to allow the binding and entry. After that, the unbound virus was removed and the cells were incubated with fresh medium (with 2% FBS). The cells and culture medium were harvested at indicated time, and subjected to western blot analysis, RT-PCR, or TCID_50_ assay, respectively.

### SDS-PAGE and western blot analysis

Cell lysates were prepared with 2 × SDS loading buffer (20 mM Tis-HCl, pH 8.0, 100 mM Dithiothreitol, 2% SDS, 20% Glycerol, and 0.016% Bromphenol blue) and denatured at 100 °C for 5 min. The whole cell lysates were separated by SDS-PAGE and transferred onto nitrocellulose membranes (Sigma-Aldrich, USA). The membranes were blocked with 5% fat free milk in Tris-buffered saline with 0.05% Tween 20 (TBST) for 1 h, incubated with the primary antibodies (1:1000 in dilution) overnight at 4 °C, then washed thrice with TBST. The membranes were then incubated with secondary antibody (1:1000 in dilution) for 1 h at room temperature and washed thrice with TBST. The protein bands were detected by enhanced chemiluminescence (ECL) detection system (Share-Bio, Shanghai, China) and exposed to Automatic chemiluminescence image analysis system (Tanon, 5200, China). After the detection, membranes were washed for 5 min with TBST, followed by rinsing with western blot stripping buffer for 20 min. Then, the membranes were rinsed with TBST and blocked with 5% fat free milk in TBST before re-probing with other antibodies.

The intensities of target bands were quantified using Image J program (NIH, USA).

### Immunofluorescence

HeLa cells were grown on 6-well chamber slides and infected with NDV. At 16 h.p.i., cells were fixed with 4% paraformaldehyde for 15 min, permeabilized with 0.5% Triton X-100 for 10 min, and blocked with 3% BSA for 30 min. The cells were incubated with antibody against CHOP or XBP1, and NDV NP (1:200 in dilution, 5% BSA) for 1 h, respectively, followed by staining with secondary antibody conjugating with FITC or TRITC (1:200 in dilution, 5% BSA) for another 1 h. Finally, cell nuclei were stained with 0.1 µg/ml of DAPI for 10 min and rinsed with PBS. The specimen was mounted with fluorescent mounting medium (DAKO) containing 15 mM NaN_3_. Images were collected with a LSM880 confocal laser-scanning microscope (Zeiss, German).

### Quantitative real-time RT-PCR

Total RNA was extracted using TRIzol^®^ Reagent (Invitrogen, USA) according to the manufacturer’s instruction. Briefly, cells were lysed with TRIzol and the lysates were mixed with one-fifth volume of chloroform. After centrifugation at 12,000 × *g* at 4 °C for 15 min, the aqueous phase was mixed with an equal volume of isopropanol. RNA was pelleted by centrifugation at 12,000 × *g* at 4 °C for 20 min, washed with 70% ethanol twice, and dissolved in RNase-free H_2_O. The concentration of the RNA was measured using a NanoDrop 2000 spectrophotometer (Thermo Fisher Scientific, USA).

cDNA was reverse transcribed from total RNA using expand reverse transcriptase (Roche, USA) and oligo-dT primer. Equal volume of cDNA was PCR-amplified using SYBR Green qPCR Mix in a CFX96TM real-time PCR system (Bio-Rad, USA). Primers used for amplification of β-actin, NP, IRE1α, XBP1u, XBP1s, P58^IPK^, ERdj4, EDEM1, IFN-β, TNF-α, IL6, and IL8 were listed in Table 2. The mRNA levels of specific genes were calculated using β-actin as an internal reference and normalized to mock-treated controls. All assays were performed in three replicates.

**Table 2:** Primer sequences used for semi-quantitative real-time RT-PCR.

Name	Sequence (5′–3′)
β-actin F	GATCTGGCACCACACCTTCT
β-actin R	GGGGTGTTGAAGGTCTCAAA
NP F	CAACAATAGGAGTGGAGTGTCTGA
NP R	CAGGGTATCGGTGATGTCTTCT
IFN-β F	GCTTGGATTCCTACAAAGAAGCA
IFN-β R	ATAGATGGTCAATGCGGCGTC
TNF-α F	AGTGACAAGCCTGTAGCCCC
TNF-α R	TTGAAGAGGACCTGGGAGT
IL6 F	TGAAAGCAGCAAAGAGGC
IL6 R	TCAAATCTGTTCTGGAGGT
IL8 F	TCCAAACCTTTCCACCCC
IL8 R	CACAACCCTCTGCACCCA
IRE1 F	CGGGAGAACATCACTGTCCC
IRE1 R	CCCGGTAGTGGTGCTTCTTA
XBP1u F	TTGTCACCCCTCCAGAACATC
XBP1u R	TCCAGAATGCCCAACAGGAT
XBP1s F	TGCTGAGTCCGCAGCAGGTG
XBP1s R	GCTGGCAGGCTCTGGGGAAG
P58IPK F	GGCTCGGTATTCCCCTTCCT
P58IPK R	AGTAGCCCTCCGATAATAAGCAA
ERdj4 F	TGTCAGGGTGGTACTTCATGG
ERdj4 R	TCTTAGGTGTGCCAAAATCGG
EDEM1 F	CGGACGAGTACGAGAAGCG
EDEM1 R	CGTAGCCAAAGACGAACATGC

F represents forward primer, R represents reverse primer

The XBP1 splicing was checked by RT-PCR using forward primer 5′-CCAAGGGGAATGAAGTGAGGC-3′ and reverse primer 5′-AGAGTTCATTAAT GGCTTCCAG-3′, which produces un-spliced XBP1 of 335 bp and spliced XBP1 of 309 bp. The PCR products were digested with the restriction enzyme *Pst I*, cleaving XBP1u into 72 and 263 bp. The digestion products were resolved on 2.5% agarose gel to separate un-spliced and spliced XBP1.

### Statistical analysis

The statistical analysis was performed with Graphpad Prism5 software (USA). The data were expressed as means ± standard deviation (SD) of at least three independent experiments. Significance was determined with the one-way analysis of variance (ANOVA). *P* values < 0.05 were deemed statistically significant.

### **Densitometry**

The intensities of corresponding bands were quantified using Image J program (NIH) according to the manufacturer’s instruction.

## Results

### NDV infection induces apoptosis

**Fig. 1:**
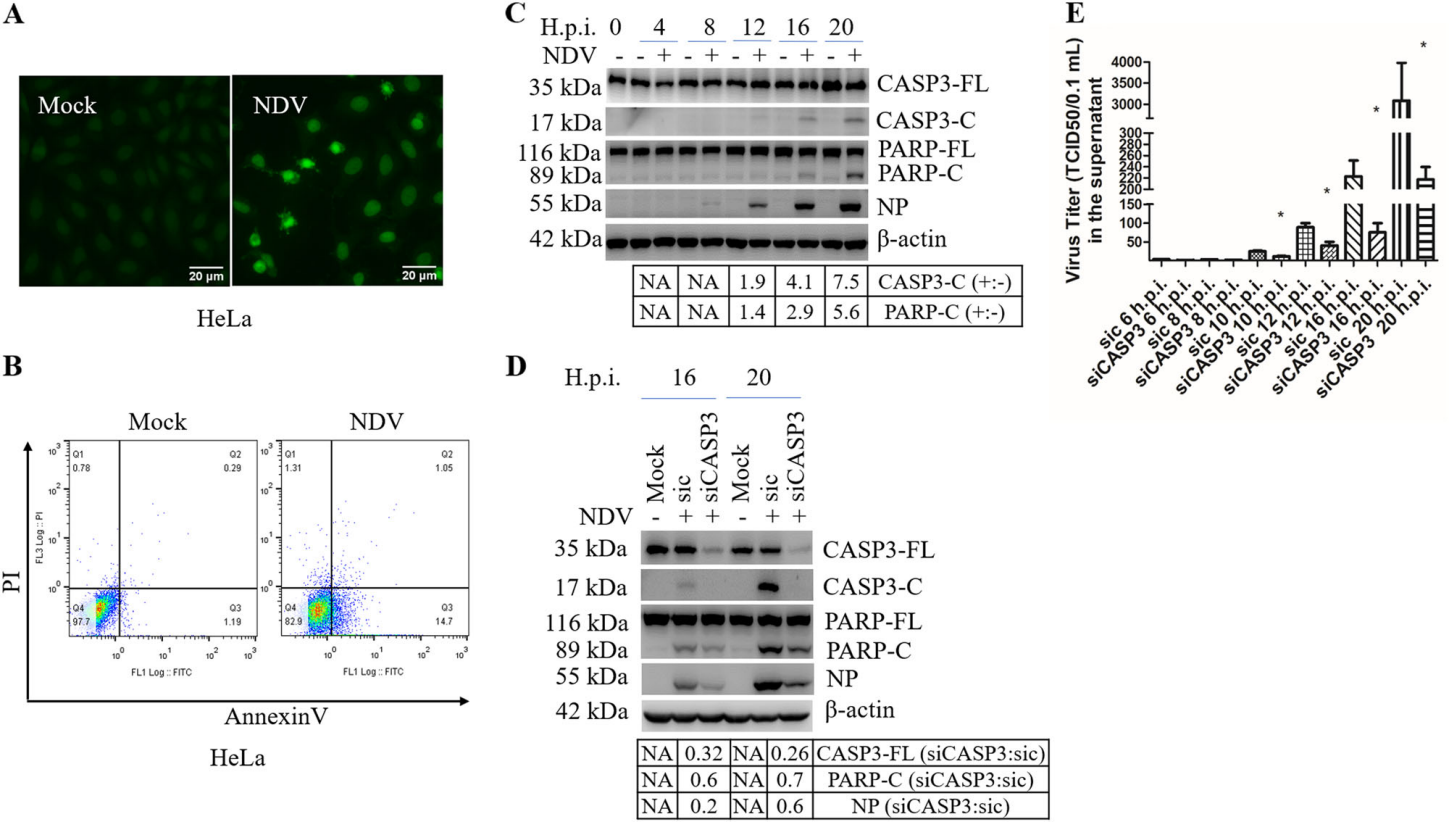
NDV infection induces apoptosis in HeLa cells. **a** Detection of NDV-induced apoptosis by TUNEL assay. HeLa cells were infected with NDV Herts/33 strain (MOI = 1) or mock-infected, and subjected to TUNEL assay at 20 h.p.i. The images of TUNEL positive cells were captured by a fluorescence microscope (×200). **b** Detection of NDV-induced apoptosis by Annexin V/PI staining and flow cytometry. HeLa cells were infected with NDV or were mock-infected, stained with annexin V and PI, and analyzed with flow cytometry at 20 h.p.i. **c** Detection of NDV-induced apoptosis in HeLa cells by western blot analysis. HeLa cells were infected with NDV or mock-infected, and harvested at 0, 4, 8, 12, 16, and 20 h.p.i. The cleavage of caspase-3 (CASP3) and PARP, the expression of NDV NP were determined by western blot analysis. The intensities of indicated protein bands were determined by image J, normalized to β-actin, and shown as fold change of NDV (+:−). **d**, **e** Knockdown of CASP3 by siRNA reduces apoptosis and virus release. HeLa cells were transfected with siCASP3 or sic for 36 h, followed by NDV infection. Mock infection without siRNA transfection was included as control. The cells lysates were analyzed with western blot with indicated antibodies (**d**). The intensities of indicated protein bands were determined, normalized to β-actin, and shown as fold change of siCASP3:sic. In parallel, the culture medium were collected at indicated time points and subjected to TCID_50_ assay (**e**). TUNEL assay, flow cytometry, and western blot are representative of three independent experiments. Virus titer represents means ± SD of three independent determinations. *p < 0.05.

**Fig. 2:**
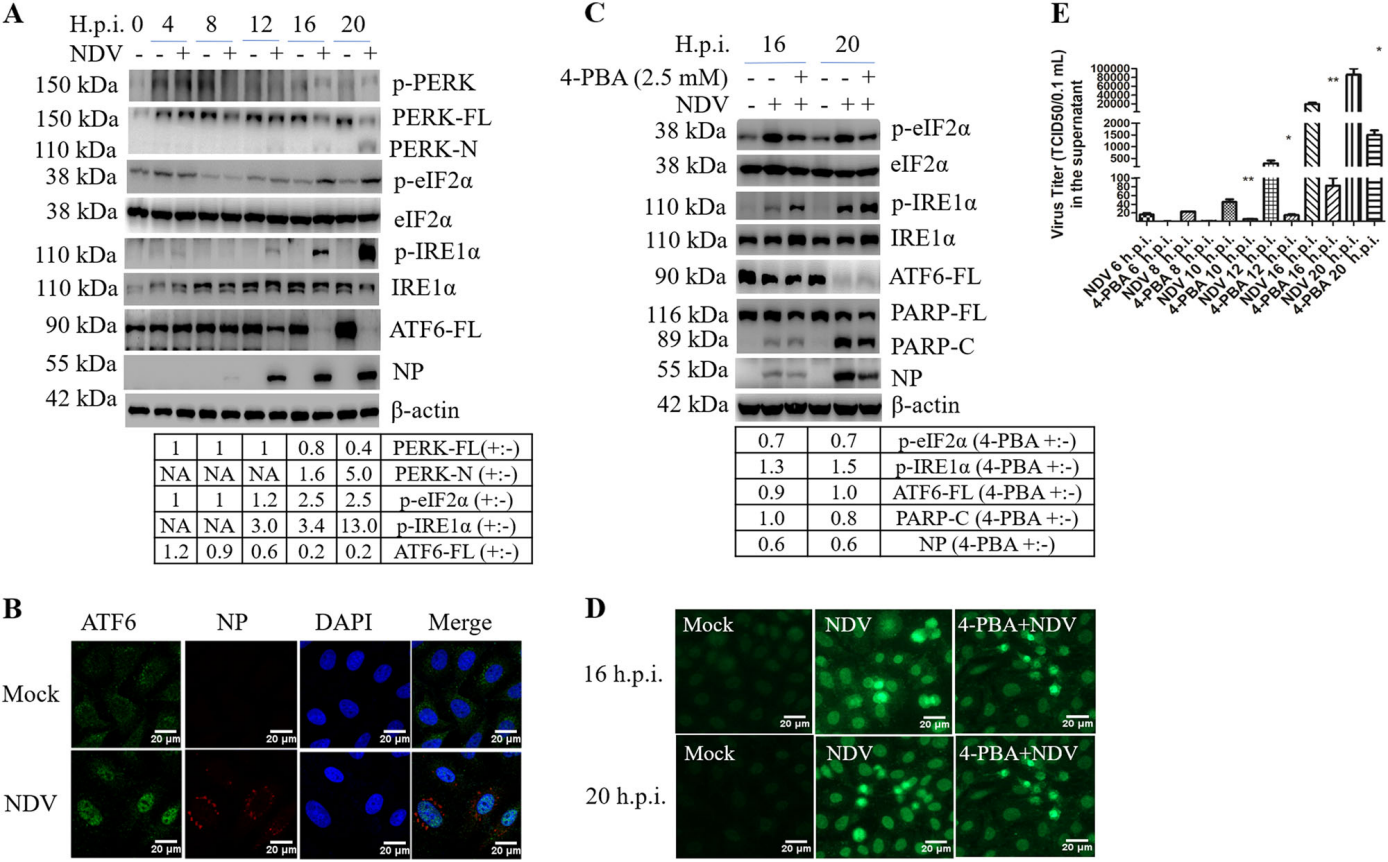
NDV infection activates three UPR signaling branches. **a** Activation of UPR pathways by NDV infection. HeLa cells were infected with NDV or mock-infected, harvested at indicated time points. The cell lysates were analyzed with western blot by using antibodies against phospho-PERK, PERK, phospho-eIF2α, eIF2α, phospho-IRE1α, IRE1α, XBP1, ATF6, NP, and β-actin. The intensities of indicated protein bands were determined, normalized to eIF2α, IRE1α, or β-actin, respectively, and shown as fold change of NDV (+:−). **b** Nuclear translocation of ATF6. HeLa cells were infected with NDV or mock-infected for 16 h, subjected to immunostaining with ATF6 and NP antibody. **c**–**e** Pharmacological inhibition of UPR suppresses virus proliferation and reduces apoptosis. HeLa cells were pretreated with 4-PBA (2.5 mM) or PBS (control) for 2 h, infected with NDV, and maintained with 2.5 mM 4-PBA during infection. Mock infection group without 4-PBA treatment was included as control. Cells were harvested at 16 and 20 h.p.i., analyzed with western blot (**c**) and TUNEL assay (**d**). In parallel, the culture medium was subjected to TCID_50_ assay, to measure the released progeny virus (**e**). The intensities of indicated protein bands were determined, normalized to eIF2α, IRE1α, or β-actin, respectively, and shown as fold change of 4-PBA (+:−). The protein bands intensities in NDV-infected cells with PBS treatment were set as 1. Western blot, Immunofluorescence, and TUNEL assay are representative of three independent experiments. Virus titer represents means ± SD of three independent determinations. *p < 0.05. **p < 0.01.

**Fig. 3:**
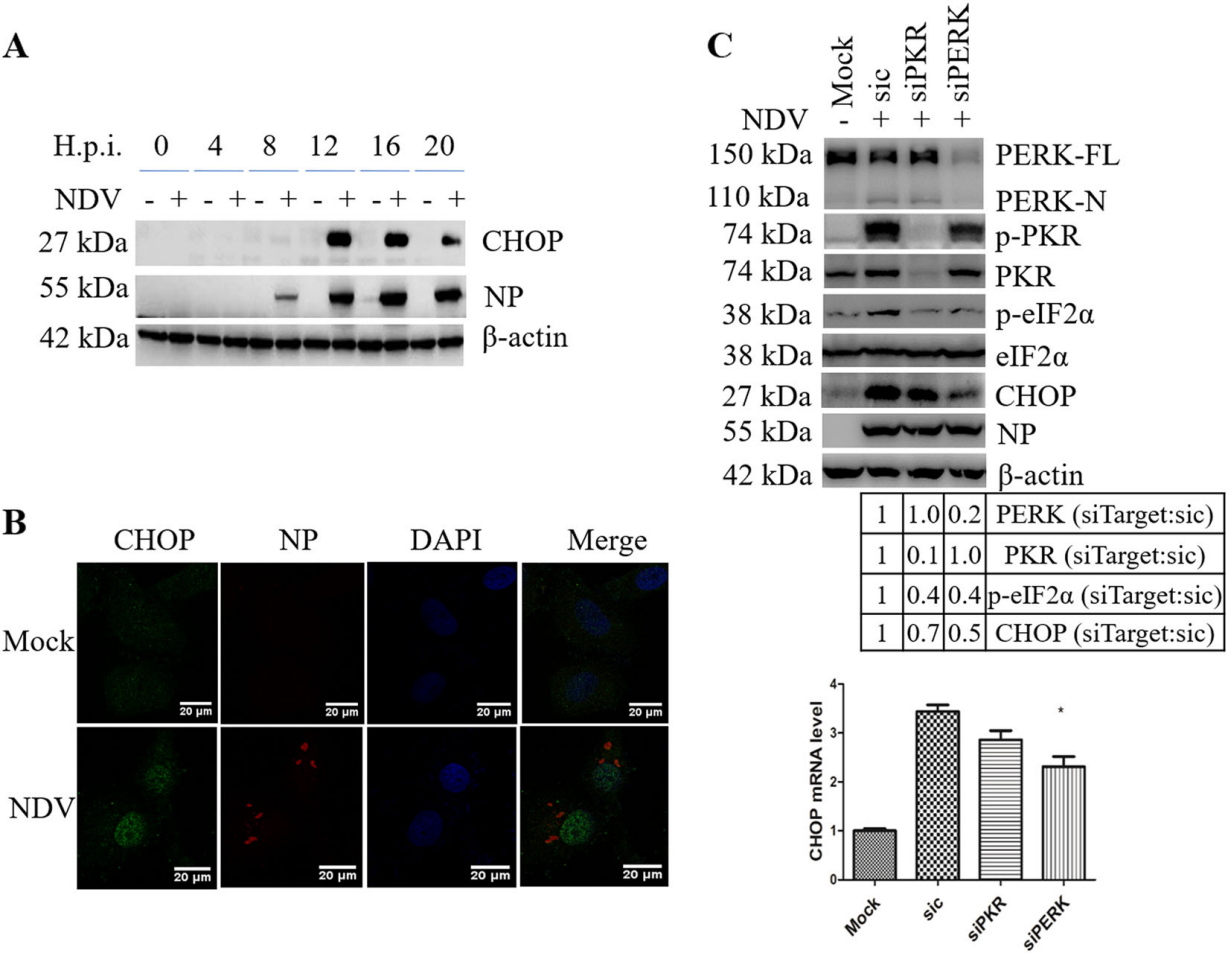
NDV infection induces the expression of pro-apoptotic transcription factor CHOP. **a** Induction of CHOP by NDV infection. HeLa cells were infected with NDV and harvested at indicated time points. The cell lysates were analyzed by western blot with antibodies against CHOP and NP. β-actin was detected as a loading control. **b** Nuclear translocation of CHOP during NDV infection. HeLa cells were infected with NDV or mock-infected for 16 h, subjected to immunostaining by using antibodies against CHOP and NP. The signal of CHOP (green) and viral protein NP (red) were observed under confocal microscope. **c** Knockdown of PKR or PERK reduces the phospho-eIF2α and expression of CHOP. HeLa cells were transfected with sic, siPERK, or siPKR, before infecting with NDV. Mock infection was set as control. At 20 h.p.i., cell samples were analyzed by western blot (upper panel) and quantitative real-time RT-PCR (low panel). The intensities of indicated protein bands were normalized to eIF2α or β-actin, respectively, and shown as fold change of siTarget:sic. Western blot and Immunofluorescence are representative of three independent experiments. CHOP mRNA level represents means ± SD of three independent determinations. *p < 0.05, ***p < 0.001.

**Fig. 4:**
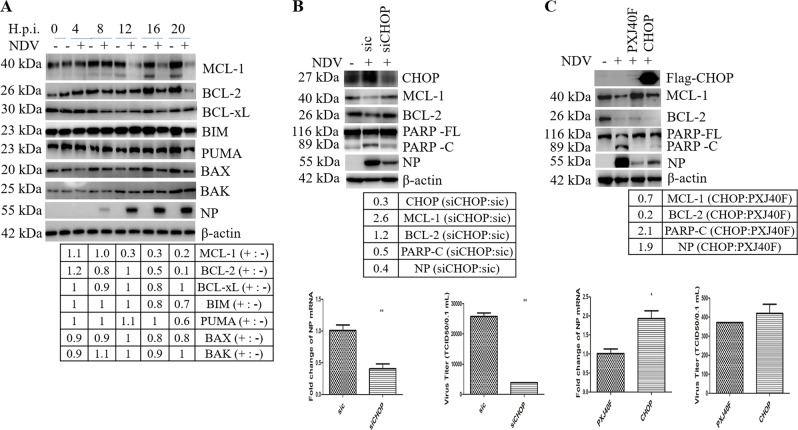
CHOP promotes apoptosis by downregulation of anti-apoptotic protein BCL-2 and MCL-1 during NDV infection. **a** Downregulation of BCL-2 and MCL-1 by NDV infection. HeLa cells were infected with NDV and harvested at indicated time points. Western blot analysis was performed to detect MCL-1, BCL-2, BCL-xL, BIM, PUMA, BAX, BAK, NP, and β-actin. The intensities of indicated protein bands were normalized to β-actin and shown as fold change of NDV (+:−). **b**, **c** CHOP regulates the levels of BCL-2/MCL-1, apoptosis, and virus proliferation. HeLa cells were transfected with sic or siCHOP for 36 h, or transfected with PXJ40F-CHOP or PXJ40F for 24 h, followed with NDV infection. Mock infection was set as control. Cells were harvested at 20 h.p.i. and analyzed with western blot (**b**, c, upper panels), or subjected to quantitative real-time RT-PCR to detect NP mRNA (**b**, **c**, low panels). In parallel, the cell culture medium was collected and subjected to TCID_50_ assay to measure the released progeny virus (**b**, **c**, low panels). The intensities of indicated protein bands were normalized to β-actin and shown as fold change of siCHOP:sic or CHOP:PXJ40F. Western blot results are representative of three independent experiments. NP mRNA level and virus titer represent means ± SD of three independent determinations. *p < 0.05, **p < 0.01.

**Fig. 5:**
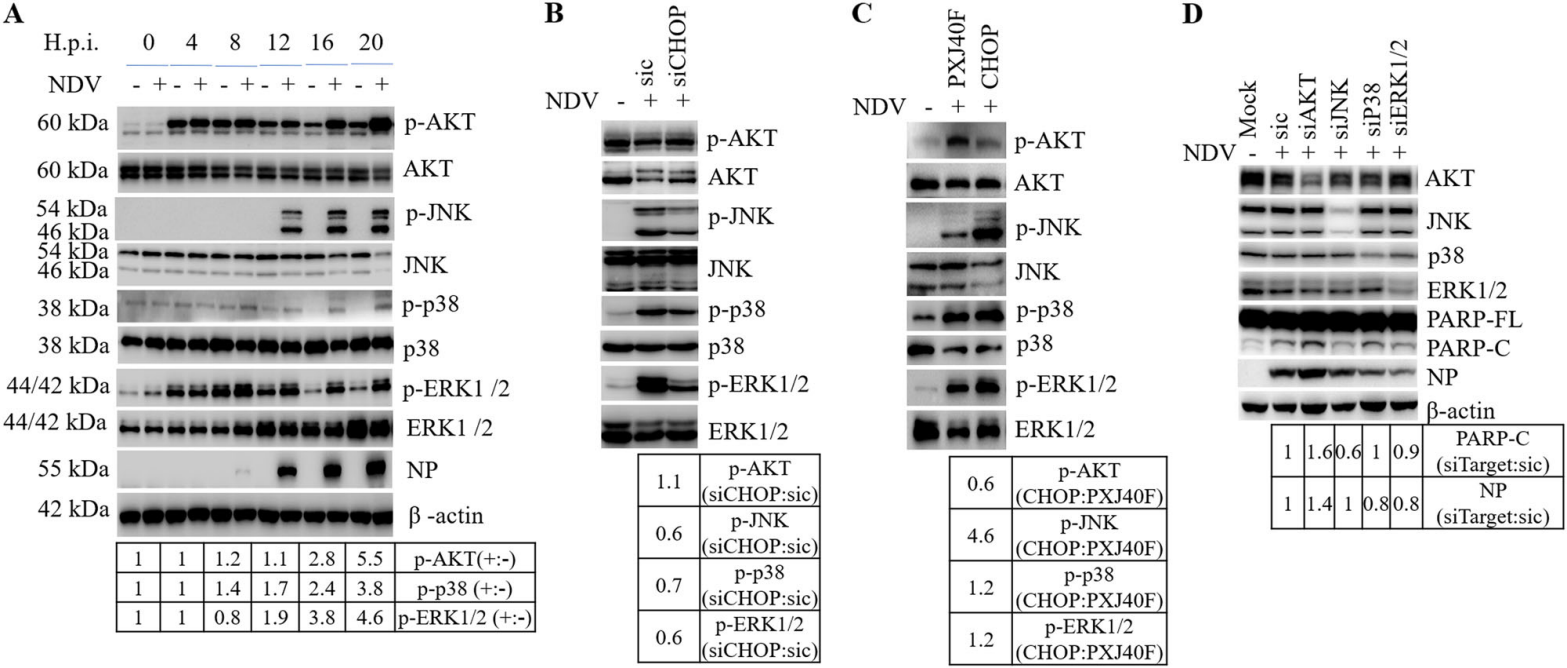
CHOP suppresses AKT signaling cascade and promotes JNK/p38 signaling cascades. **a** Activation of AKT, JNK, p38, and ERK1/2 signaling cascades during NDV infection. HeLa cells were infected with NDV and harvested at indicted time points. Western blot analysis was performed using antibodies against phospho-AKT, AKT, phospho-JNK, JNK, phospho-p38, p38, phospho-ERK1/2, ERK1/2, NP, and β-actin. The intensities of phospho-AKT, phospho-JNK, phospho-p38, and phospho-ERK1/2 bands were normalized to corresponding total proteins and shown as fold change of NDV (+:−). **b**, **c** CHOP regulates the AKT signaling cascades and suppresses MAPK signaling cascades. Cell lysates prepared in Fig. 4b, c were subjected to western blot with indicated antibodies. The intensities of phospho-AKT, phospho-JNK, phospho-p38, and phospho-ERK1/2 bands were normalized to corresponding total proteins, shown as fold change of siCHOP:sic or CHOP:PXJ40F. Results shown are representative of three independent experiments. **d** The effect of knockdown of AKT, JNK, p38, or ERK1/2 on NDV-induced apoptosis and virus proliferation. HeLa cells were transfected with sic, siAKT, siJNK, sip38, or siERK1/2, then infected with NDV for 20 h. Mock infection was included as control. Cell lysates were analyzed by western blot with indicated antibodies. The intensities of PARP-C and NP were normalized to β-actin and shown as fold change of siTarget:sic. Results shown are representative of three independent experiments.

**Fig. 6:**
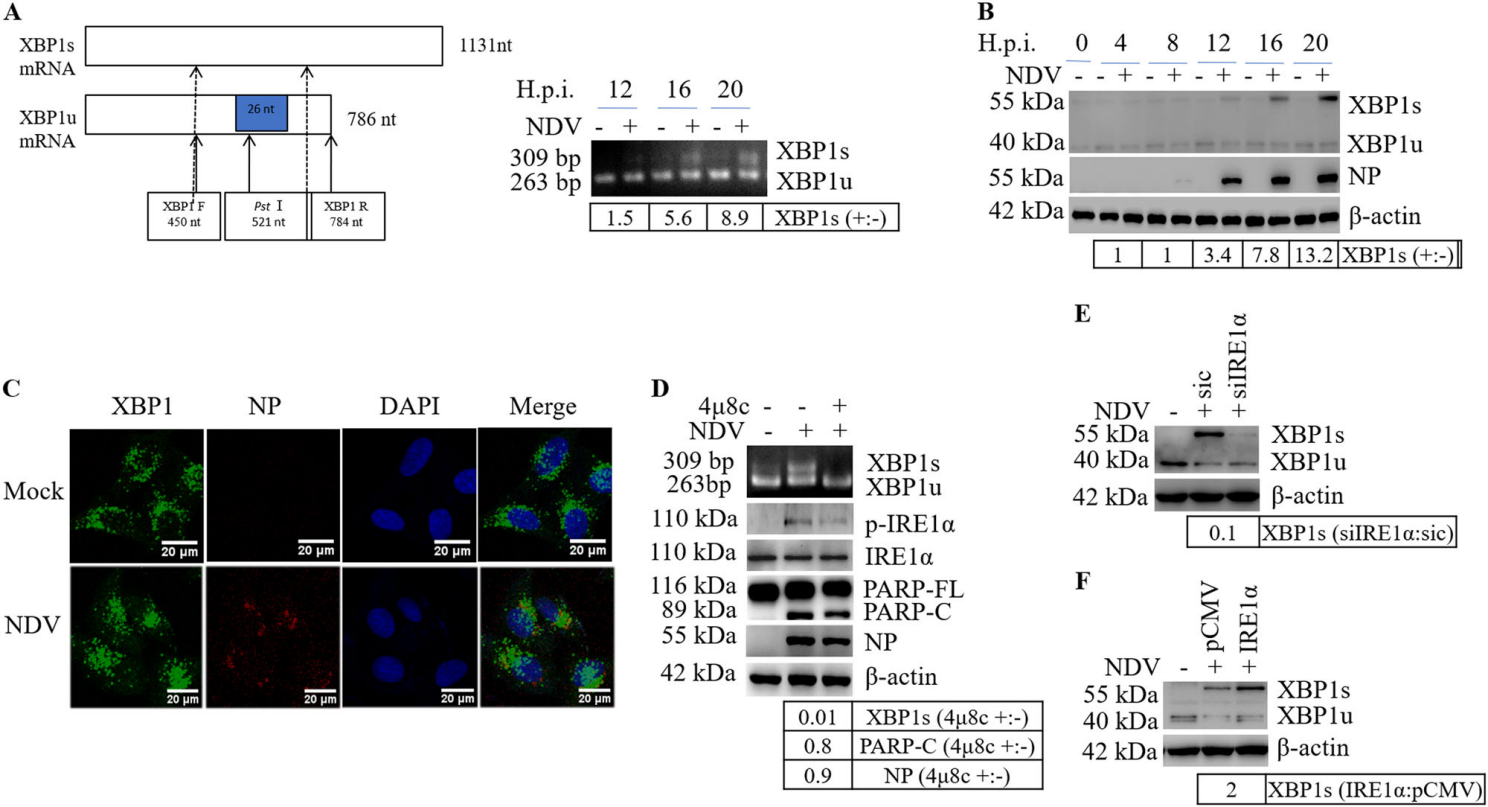
NDV infection triggers the activation of IRE1α-XBP1 signaling. **a** NDV infection promotes the splicing of XBP1 mRNA into XBP1s. HeLa cells were infected with NDV or mock-infected and were analyzed with RT-PCR to detect the spliced form of XBP1 mRNA. RT-PCR was performed with XBP1 specific primers and the products were digested with *Pst I*. XBP1u products were 72 bp and 263 bp, XBP1s product was 309 bp. The 309 bp of XBP1s and 263 bp of XBP1u were shown in the agarose gel. The intensities of XBP1s 309 bp band were shown as fold change of NDV (+:−). **b** NDV infection promotes the expression of XBP1s. HeLa cells were infected with NDV and subjected to western blot to detect XBP1u and XBP1s. The intensities of XBP1s were normalized to β-actin and shown as fold change of NDV (+:−). **c** The nuclear translocation of XBP1 during NDV infection. HeLa cells were infected with NDV or mock-infected for 16 h, then subjected to immunofluorescence to detect XBP1 and NP. **d** Inhibition of IRE1α RNase activity by 4µ8c blocks XBP1 mRNA splicing. HeLa cells were infected with NDV, treated with DMSO or 25 µM IRE1α inhibitor 4µ8c, and subjected to RT-PCR by using XBP1 specific primers. The products were digested with *Pst I*, and the 309 bp of XBP1s mRNA and 263 bp of XBP1u mRNA were shown in agarose gel. In parallel, western blot analysis was performed to check the effect of 4µ8c on PARP cleavage and NDV NP protein expression. The intensities of XBP1s, PARP-C, NP bands were determined, shown as fold change of 4µ8c (+:−). **e**, **f** IRE1α mediates XBP1 splicing. HeLa cells were transfected with sic or siIRE1α for 36 h (**e**), or transfected with plasmid pCMV or pCMV-IRE1α for 24 h (**f**), followed with NDV infection. The cells were harvested at 20 h.p.i. and analyzed with western blot by using XBP1 antibody. The intensities of XBP1s bands were determined and normalized to β-actin, and shown as fold change of siIRE1α:sic, or IRE1α:pCMV. Results are representative of three independent experiments.

**Fig. 7:**
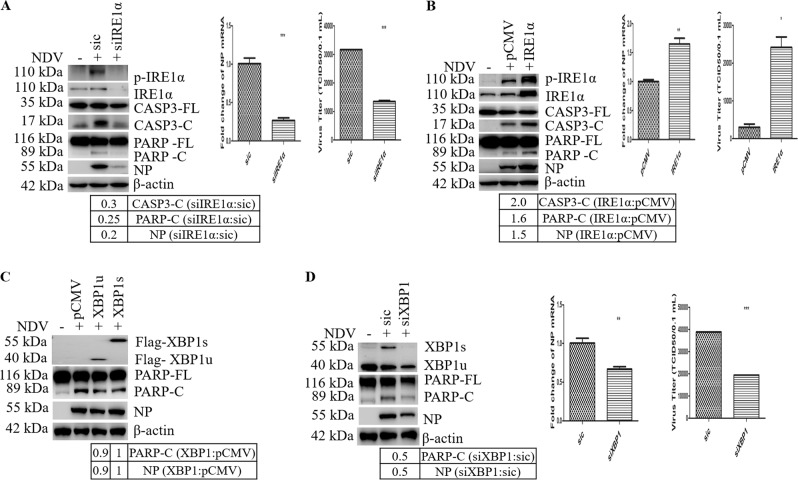
IRE1α-XBP1 promotes apoptosis and NDV proliferation. **a**, **b** IRE1α promotes apoptosis and NDV proliferation. The cell lysates in Fig. 6e, f were analyzed with western blot with antibodies against phospho-IRE1α, IRE1α, CASP3, PARP, NP, and β-actin. The intensities of CASP3-C, PARP-C, and NP bands were normalized to β-actin and shown as fold change of siIRE1α:sic or IRE1α:pCMV. Meanwhile, the samples were analyzed with quantitative real-time RT-PCR to detect NP mRNA. The virus titers in culture medium were determined with TCID_50_ assay. mRNA and virus titer data represent means ± SD of three independent determinations. *p < 0.05, **p < 0.01; ***p < 0.001. **c**, **d** XBP1 is involved in apoptosis and NDV proliferation. HeLa cells were transfected with plasmid pCMV-XBP1u, pCMV-XBP1s, or pCMV, or transfected with sic or siXBP1, followed with NDV infection for 20 h. Mock infection without any transfection was included as control. Cell lysates were prepared and blotted. The intensities of PARP-C and NP bands were normalized to β-actin, and shown as fold change of XBP1:pCMV or siXBP1:sic. Results are representative of three independent experiments.

**Fig. 8:**
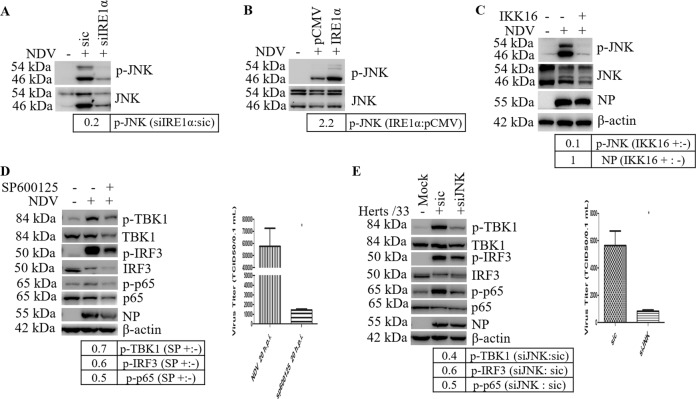
NDV infection activates pro-apoptotic and pro-inflammatory JNK signaling cascade via IRE1α and NF-κB pathway. **a**, **b** IRE1α promotes JNK signaling. Cell samples in Fig. 6e, f were analyzed with western blot to check the phospho-JNK and JNK. The intensities of phospho-JNK bands were normalized to total JNK, shown as fold change of siIRE1α:sic or IRE1α:pCMV. **c** Pharmacologic inhibition of NF-κB signaling suppresses JNK activation. HeLa cells were infected with NDV, followed by incubation with DMSO or 5 µM IKK16. At 20 h.p.i., the levels of phospho-JNK, JNK, NP, and β-actin were analyzed with western blot. The intensities of phospho-JNK and NP bands were normalized to total JNK or β-actin, shown as fold change of IKK16 (+:−). **d**, **e** Inhibition of JNK suppresses IRF3 and NF-κB signaling and reduces virus proliferation. HeLa cells were infected with NDV, followed by treatment with DMSO or 7.5 µM JNK inhibitor SP600125, and harvested at 20 h.p.i. (**d**). In parallel, HeLa cells were transfected with siJNK or sic for 48 h, followed by NDV infection for 20 h (**e**). Mock infection was included as control. The protein level of phospho-TBK1, TBK1, phospho-IRF3, IRF3, phospho-p65, p65, NP, and β-actin were analyzed with western blot. The intensities of phospho-TBK1, phospho-IRF3 and phospho-p65 bands were normalized to total TBK1, IRF3, p65, shown as fold change of SP600125 (+:−) or siJNK:sic. Western blot shown are representative of three independent experiments. Meanwhile, the cell culture supernatants were collected and subjected to TCID_50_ assay. Virus titer represent means ± SD of three independent determinations. *p < 0.05.

The pathogenesis and oncolytic activity of NDV are associated with cell death. To study whether NDV induces apoptosis in host cells and human cancer cells, chicken cells CEF and DF-1, human cancer cells HeLa, Cal27, HN13, A549, H1299, Huh7, and HepG2, were infected with Herts/33, a virulent strain of NDV. These infected cells were then subjected to apoptosis analysis. The non-cancerous human embryonic kidney cells, 293T, were infected as a control cell type. Due to space limitations, we presented the results from HeLa cells in Figs. 1–8 and the results for other cell lines in supplementary figures. As expected, at 20 h.p.i., increased number of TUNEL positive cells was observed in HeLa (Fig. 1a), as well as in CEF, DF-1, Cal27, HN13, A549, H1299, Huh7, and HepG2 cells (Fig. S1A); only few TUNEL positive cells were observed in 293T cells (Fig. S1A). Annexin V/PI staining confirmed that NDV indeed induced functional apoptosis in chicken and human cancer cells, but not in 293T cells (Fig. 1b, Fig. S1B). Immunoblotting showed that in HeLa, Cal27, HN13, A549, and H1299 cells, both major biochemical markers of apoptosis, caspase-3 and PARP, were cleaved into smaller fragments in an infection time-dependent manner; in Huh7 and HepG2 cells, weak cleavage of PARP appeared at 24 h.p.i.; no cleavage of both biochemical markers was observed in 293T cells (Fig. 1c and Fig. S1C). Due to unavailabilbty antibodies, chicken cells were not included in the immunoblotting analysis. These observations suggest that NDV infection triggers functional apoptosis in chicken cells and many human cancer cell types, but not in non-cancerous 293T cells.

To investigate the role of apoptosis in NDV proliferation, we utilized siRNA to specifically knockdown caspase-3. As shown in Fig. 1d, caspase-3 was successfully knocked down, accompanying with decreased cleavage of PARP. Interestingly, the expression of viral NP was also reduced at late infection time points. Examination of extracellular virus titer showed that knock down of caspase-3 significantly suppressed the production of progeny virus (Fig. 1e). These results suggest that apoptosis promotes virus proliferation, probably by facilitating virus release and spread.

### NDV infection activates the three branches of UPR signaling

We next set up to adress if the UPR is involved in NDV-induced apoptosis. First, we examined the three branches of UPR in NDV-infected cells. As shown in Fig. 2a and Fig. S2, at late infection time points. Interestingly, the full-length PERK (PERK-FL) was cleaved into a 110-kDa N-terminal fragment (PERK-N) in HeLa, Cal27, HN13, A549, and H1299, but not in Huh7, HepG2, and 293T cells. The cleavage site of PERK was mapped to Thr980 (data not shown). In the mock-infected group, there was basal level of phospho-PERK, probably due to the removal of serum in the culture medium (Fig. 2a and Fig. S2). The phospho-PERK was clear observed in NDV-infected Hela cells at 16 and 20 h.p.i. (Fig. 2a), however, in other cancer cell lines, the level of phospho-PERK in NDV-infected cells was lower than or comparable to that in mock-infected cells, probably due to the cleavage of PERK-FL or other unknown mechanisms (Fig. S2). Meanwhile, phospho-eIF2α was gradually increased at late infection time points in HeLa cells (Fig. 2a), and in HN13, A549, Huh7, HepG2, and 293T cells (Fig. S2). The failure to observe the increase of phospho-eIF2α in NDV-infected Cal27 and H1299 cells might be attributed to the high basal level of phospho-eIF2α in mock-infected cells. These results demonstrate that NDV infection induces the phosphorylation of eIF2α in various cancer cells; however, the cleavage of PERK may attenuate the activation of PERK-eIF2α branch. Meanwhile, NDV infection greatly stimulated the phosphorylation of IRE1α in HeLa (Fig. 2a), Cal27, HN13, A549, H1299, Huh7, and HepG2 cells, but not in 293T cells (Fig. S2). The level of full-length ATF6 (ATF6-FL) was gradually decreased at late infection time in HeLa (Fig. 2a), Cal27, A549, H1299, Huh7, and 293T, but not in HN13 and HepG2 (Fig. S2). Although we failed to detect the cleavage band of ATF6, the nuclear translocation of ATF6 was indeed observed in NDV-infected HeLa cells (Fig. 2b). These results demonstrate that NDV infection triggers the eIF2α, IRE1α, and ATF6 signaling in most cancer cell types studies.

We next evaluated the role of UPR in apoptosis and virus proliferation, by utilizing a chemical chaperone 4-PBA to attenuate ER stress^39^. In HeLa cells receiving both 4-PBA and NDV, the level of phospho-eIF2α was reduced by 0.7-fold; interestingly, both phospho-IRE1α and total IRE1α were increased, whereas the cleavage of ATF6-FL was not affected, compared to those cells receiving NDV only (Fig. 2c). Thus, 4-PBA only suppressed the phosphorylation of eIF2α in NDV-infected cells. Further study showed that 4-PBA treatment decreased the cleavage of PARP and produced less TUNNEL positive cells (Fig. 2c, d), accompany with lower expression of NP and less release of progeny virus (Fig. 2e). Thus, alleviation of eIF2α phosphorylation by 4-PBA may protect cells from death and is not favorable for virus proliferation.

### NDV infection induces the expression of CHOP via PERK and PKR signaling

Under prolonged ER stress, the preferentially translation of ATF4 usually promotes the expression of pro-apoptotic transcription factor CHOP^40,41^. Here, we found that the expression of CHOP was significantly induced at late infection time in all cell types (Fig. 3a, Fig. S3A). Immunofluorescence showed that strong CHOP signal was mainly localized in the nucleus at 16 h.p.i. (Fig. 3b). Thus, the persistent exposure to NDV infection greatly induces the expression and nuclear translocation of CHOP.

To clarify whether PERK-eIF2α or PKR-eIF2α signaling is involved in CHOP induction, we specifically knocked down PERK and PKR, respectively. Results showed that knock down of either PKR or PERK decreased the level of phospho-eIF2α and reduced the expression of CHOP at both mRNA and protein level (Fig. 3c). Pharmacological inhibition of PERK and PKR by 10 μM GSK2606414 confirmed above results (Fig. S3B). Thus, both PERK and PKR contribute to the phosphorylation of eIF2α and transcriptional induction of CHOP during NDV infection.

### CHOP promotes apoptosis by reducing the level of anti-apoptotic protein BCL-2 /MCL-1

Is CHOP involved in NDV-induced intrinsic apoptosis? It has been known that CHOP promotes mitochondria mediated apoptosis via downregulation of the pro-survival BCL-2^24,42^. First, we examined the levels of BCL-2 family members during NDV infection. As shown in Fig. 4a, pro-survival guardian protein BCL-2 and MCL-1 were gradually decreased from 16 to 20 h.p.i., while BCL-xL remained relatively stable; the pro-apoptotic BIM and PUMA, the pore forming protein BAX, were slightly decreased at 16 and 20 h.p.i., and the pore forming protein BAK was kept at a steady level. The decrease of BCL-2/MCL-1 may allow BAX/BAK to form pores in the outer membranes of mitochondria.

To investigate whether CHOP is involved in regulating the levels of BCL-2 and MCL-1, we specifically knocked down the expression of CHOP, followed with NDV infection. As shown in Fig. 4b, accompanying with depletion of CHOP, the levels of MCL-1 and BCL-2 were recovered, and the cleavage of PARP was reduced; interestingly, significant decrease of the expression of NP and the release of progeny virus were observed. To further demonstrate above observation, we adopted the transient overexpression approach. As shown in Fig. 4c, compared with vector-transfected group, overexpression of CHOP greatly reduced the levels of MCL-1 and BCL-2, resulting in higher level of PARP cleavage; the level of viral protein NP and the release of progeny virus were greatly increased. These data confirm that induction of CHOP promotes apoptosis via downregulating BCL-2/MCL-1 and supports NDV proliferation.

### CHOP promotes apoptosis by regulating AKT and JNK signaling cascades

AKT plays a critical role in promoting cell survival^43^. MAPK cascades are involved in cell growth, differentiation, and control of cellular responses to cytokines and stress^44,45^. To investigate whether AKT and MAPK signaling are involved in NDV-induced apoptosis, the kinetic activation of these kinases was examined. As shown in Fig. 5a, although there were basal levels of phospho-AKT and phospho-ERK1/2 after the serum was removed from culture medium during infection, the levels of both phospho-AKT and phospho-ERK1/2 were increased at late infection time; meanwhile, a gradual increase of phospho-JNK and phospho-p38 were clearly detected. These observations reveal that NDV infection activates AKT and three branches of MAPK signaling.

To study whether CHOP is involved in regulating AKT and MAPK signaling, we examined the phosphorylation levels of these kinases by knock down or overexpression of CHOP. As shown in Fig. 5b, depletion of CHOP indeed decreased the levels of phospho-JNK, phospho-p38, and phospho-ERK1/2 by 0.6- to 0.7-fold, respectively, although the level of phospho-AKT was not changed. In contrast, overexpression of CHOP reduced the level of phospho-AKT by 0.6-fold and greatly stimulated phosphorylation of JNK by 4.6-fold, phosphorylation of p38 and ERK1/2 by 1.2-fold (Fig. 5c). From these data, we speculate that CHOP may promote apoptosis by suppressing AKT signaling and enhancing MAPK signaling.

To evaluate the effect of AKT and MAPK signaling on apoptosis, we employed siRNA to specifically interfere the expression of AKT, JNK, p38, or ERK1/2. As shown in Fig. 5d, knock down of AKT obviously increased the cleavage of PARP; knocking down of JNK slightly decreased the cleavage of PARP; depletion of p38 and ERK1/2 did not significantly change the cleavage of PARP. These observations were further confirmed by pharmacological inhibition of AKT, JNK, p38, and ERK1/2, respectively (Fig. S4A–D). Thus, AKT and JNK are the major pathways involved in apoptosis during NDV infection.

### IRE1α mediated-XBP1 splicing is essential for efficient NDV proliferation and apoptosis

We have observed the activation of IRE1α in Fig. 2 and Fig. S2, next, we examined the splicing of XBP1. As shown in Fig. 6, the splicing form of XBP1s mRNA was clearly detected at 12–20 h.p.i. (Fig. 6a), meanwhile, the 55 kDa XBP1s protein was observed (Fig. 6b). The expression of XBP1s was also detected in NDV-infected Cal27, HN12, A549, H1299, Huh7, HepG2, and 293T cells (Fig. S2). Immunofluorescence showed that XBP1 was translocated to the nucleus in response to NDV infection (Fig. 6c). These results clearly demonstrate that NDV infection triggers XBP1s mRNA splicing and produces XBP1s as a nuclear transcription factor.

We next examined the effect of IRE1α on XBP1 splicing. IRE1α ribonuclease activity was inhibited by 4μ8c, which specifically binds to the lysine residue in the catalytic pocket. Results in Fig. 6d showed that 25 μM 4μ8c treatment markedly suppressed NDV-induced splicing of XBP1 mRNA. To eliminate the possibility of off target by chemical treatment, we specifically knocked down or overexpressed IRE1α. Compared with control group, knock down of IRE1α reduced the level of XBP1s to undetectable level (Fig. 6e); in contrast, overexpression of IRE1α produced 2-fold of XBP1s (Fig. 6f). Collectively, these results confirm that IRE1α ribonuclease is responsible for the splicing of XBP1 mRNA and production of XBP1s in response to NDV infection.

### IRE1α-XBP1 promotes NDV-induced apoptosis and facilitates viral proliferation

IRE1α has shown to be involved in cell death under prolonged/severe ER stress^25,46^. To assess the role of IRE1α on cell fate during NDV infection, IRE1α was knocked down. As shown in Fig. 7a, compared to those in sic-transfected cells, knock down of IRE1α reduced the cleavage of caspase-3 and PARP, impaired the expression of NP and the production of progeny virus. Meanwhile, the transcriptional induction of the ER chaperones and components of ERAD, including p58^IPK^, ERdj4, and EDEM1 genes, was reduced in IRE1α knock down cells (Fig. S5A). We further analyzed this observation by overexpression of IRE1α. As shown in Fig. 7b, compared with vector pCMV trasfected cells, overexpression of IRE1α promoted the phosphorylation of IRE1α, enhanced the cleavage of caspase-3 and PARP, and increased the expression of NP and production of progeny virus. As expected, the transcription of the ER chaperones and ERAD components was increased (Fig. S5B).

To study the role of XBP1 in NDV-induced apoptosis, HeLa cells were transfected with Flag-tagged XBP1u, XBP1s, and pCMV vector. Neither XBP1u nor XBP1s changed the cleavage of PARP and the synthesis of NP (Fig. 7c). However, knock down of XBP1 reduced the cleavage of PARP and suppressed virus proliferation, as evidenced by the decrease of NP synthesis, viral mRNA transcription, and progeny virus production (Fig. 7d). These results demonstrate that IRE1α-XBP1 triggers the transcription of ER chaperones/ERAD components, promotes apoptosis, and supports efficient NDV replication.

### JNK signaling is activated via IRE1α/NF-κB signaling and is involved in NDV-induced apoptosis/inflammation

We next asked whether IRE1α is involved in JNK activation. Interfering the expression of IRE1α by siRNA reduced the level of phospho-JNK by 0.2-fold (Fig. 8a); in contrast, in cells transfected with plasmid encoding IRE1α, the level of phospho-JNK was markedly increased by 2.2-fold (Fig. 8b). These data demonstrate that IRE1α promotes JNK phosphorylation in NDV-infected cells. Previously, we reported that NDV infection-induced death ligand TNF-α expression via NF-κB pathway^37^. Usually, the binding of TNF-α to TNFR not only activates caspase 8 and NF-κB, but also triggers JNK^47^. To examine whether NF-κB signaling is involved in the activation of JNK, IKKβ inhibitor IKK16 (5 μM) was incubated with NDV-infected cells to block the activation of NF-κB. As shown in Fig. 8c, inhibition of NF-κB signaling reduced the level of phospho-JNK to undetectable level, demonstrating that JNK is also activated via the NF-κB pathway.

JNK promotes apoptosis as well as inflammation^48^. Is the activation of JNK involved in the transcriptional induction of cytokines? Here, we used SP600125 to inhibit JNK kinase activity, or specifically interfered the expression of JNK by siRNA, then evaluated IRF3 and NF-κB signaling. Results in Fig. 8d, e showed that both inhibition and knocking down of JNK reduced the levels of phospho-TBK1, phospho-IRF3 and phospho-p65; surprisingly, virus proliferation was also suppressed. Accordingly, the transcription of TNF-α, IL6, and IL8 was markedly suppressed; the transcription of IFN-β was significantly suppressed by the inhibitor treatment, but only slightly decreased in JNK knock down cells (Fig. S5A, B). Collectively, these results demonstrate that JNK promotes cytokines transcription during NDV infection, probably via regulation of NF-κB and IRF3 signaling. It was worthwhile to note that the release of progeny virus was greatly suppressed by inhibition of JNK, suggesting that JNK signaling is helpful for virus proliferation.

## Discussion

As an acute infection pathogen and oncolytic reagent, NDV specifically kills host cells and tumor cells by inducing apoptosis and inflammation. In this study, we demonstrate that NDV infection initiates the UPR signaling and triggers functional apoptosis in chicken cells as well as in various cancer cell types; suppression of either UPR or apoptosis is not favorable for efficient NDV proliferation. Indeed, to facilitate shedding and dissemination of progeny viruses, some viruses take advantage by initiating apoptosis^49^. Apoptosis may help NDV release by killing the infected cells, promote virus spread to neighbor cells by avoiding stimulation of anti-viral innate immune responses or inflammation in un-infected neighbor cells, thereby quickly establishing acute infection.

UPR determines cell fate to survival or death^50,51^. Here, we find that the UPR is involved in NDV-induced apoptosis by triggering the expression of CHOP via PERK/PKR-eIF2α dependent manner. The mechanisms of CHOP to promote apoptosis include decreasing the levels of BCL-2 and MCL-1, which may result in release of BAX to form pores in mitochondria outer membranes^52^; limiting the activation of pro-survival AKT; and promoting the signaling of pro-apoptotic JNK^53,54^. Activation of p38 during NDV infection may also lead to phosphorylation and activation of CHOP^55,56^. Interestingly, induction of CHOP supports efficient NDV proliferation.

IRE1α is a highly conserved ER stress sensor, which is involved in determination of cell fate^57^. Viruses have different mechanisms to regulate IRE1α signaling, to facilitate their own replication. Hepatitis B virus, Influenza A virus, Japanese encephalitis virus, and Flavivirus activate IRE1-XBP1 singaling^58–61^, while Hepatitis C virus and Rotavirus suppress this pathway^62,63^. In this study, we demonstrate that NDV infection activates IRE1α-XBP1 and IRE1α-JNK signaling, leading to sensitizing cells to apoptosis and enhancing NDV proliferation. JNK also promotes the expression of pro-inflammatory cytokines. The activation of JNK is not only modulated by CHOP and IRE1α, but also triggered by NF-κB signaling. Thus, JNK plays an essential role in the crosstalk of UPR, apoptosis, and inflammation during NDV infection.

The principal findings of this study are NDV infection promotes apoptosis and inflammation in various cancer cell types via UPR, including the eIF2α-CHOP-BCL-2/JNK signaling and IRE1α-XBP1s/JNK signaling, which is helpful for NDV proliferation (summarized in Fig. S7). The full understanding of the involvement of these UPR branches in NDV replication process appears to be complicated. In addition to helping NDV spread to neighbor cells by triggering apoptosis, the global translation shut off caused by PERK/PKR-eIF2α signaling may allow the translational machinery hacked by virus and translate the viral proteins preferentially. Alternatively, the expression of ER quality control proteins, which are controlled by IRE1α-XBP1 pathway, promotes virus replication by enhancing the viral proteins modification, folding, and trafficking. Another possibility is that XBP1s stimulates the phospholipid biosynthesis and ER expansion^64^, providing the lipid that is necessary for the enveloped virus particle assembly. Activation of JNK by UPR not only contributes to apoptosis, but also initiates inflammation by promoting the transcription of cytokines. The secretion of cytokines may attract the phagocyte to engulf the infected-apoptotic cells. In all, this study provides comprehensive insights into the mechanisms of UPR induced apoptosis and cytokinesecretion during NDV infection.

## Supplementary information

Supplmentary figure legend

Figure S1

Figure S2

Figure S3

Figure S4

Figure S5

Figure S6

Figure S7
