# Supplementary material for: eIF2α-CHOP-BCl-2/JNK and IRE1α-XBP1/JNK signaling promote apoptosis and inflammation and support the proliferation of Newcastle disease virus
Source: Cell Death Dis. 2019 Nov 26;10(12):891. doi: 10.1038/s41419-019-2128-6 (PMC6877643; doi:10.1038/s41419-019-2128-6)
Supplement: Supplementary file 1 — Supplmentary figure legend [file 41419_2019_2128_MOESM1_ESM.docx]

**Figure S1. NDV infection induces apoptosis in various cell in chicken cells and human cancer cells.** (A) Detection of NDV-induced apoptosis by TUNEL assay. CEF, DF1, Cal27, HN13, A549, H1299, Huh7, HepG2, and 293T were infected with NDV and harvested at 20 h.p.i.. Mock infection was included as negative control. TUNEL assay was carried out and the images of TUNEL positive cells were captured by a fluorescence microscope (200×). (B) Detection of NDV-induced apoptosis by Annexin V/PI staining and Flow Cytometry. CEF, DF1, Cal27, HN13, A549, H1299, Huh7, HepG2, and 293T cells were infected with NDV and harvested at 20 h.p.i.. Mock infection was included as negative control. Cell were stained with Annexin V and PI, followed with Flow Cytometry analysis. (C) Detection of NDV-induced apoptosis by Western blotting analysis. Cal27, HN13, A549, H1299, Huh7, HepG2, and 293T cells were infected with NDV and harvested at 16, 20, and 24 h.p.i.. At each time point, mock infection was included as negative control. Cell lysates were subjected to Western blotting analysis by using antibodies against CASP3, PARP, NP, and β-actin. β-actin was detected as a loading control. Tunnel assay, Flow cytometry, and Western blot shown are representative of three independent experiments.

**Figure S2. NDV infection activates three UPR signaling branches in various cell types.** Cal27, HN13, A549, H1299, Huh7, HepG2, and 293T cells were infected with NDV and harvested at 16, 20, and 24 h.p.i. At each time point, mock infection was included as negative control. The cell lysates were analyzed with Western blotting by using antibodies against phospho-PERK, PERK, phospho-eIF2α, eIF2α, phospho-IRE1α, IRE1α, XBP1, and ATF6. Results shown are representative of three independent experiments.

**Figure S3. NDV infection induces the expression of CHOP via PERK and PKR signaling in various cell types.** (A) Cal27, HN13, A549, H1299, Huh7, HepG2, and 293T cells were infected with NDV and harvested at 16, 20, 24 h.p.i.. At each time point, mock infection was included as negative control. The cell lysates were analyzed with Western blotting by using antibodies against CHOP. Results shown are representative of three independent experiments. (B) Pharmacological inhibition of PERK and PKR activity reduces the phosphorylation of eIF2α and the expression of CHOP. HeLa cells were infected with NDV and treated with 10 μM of GSK2606414 (GSK) or DMSO (control). Cell samples were harvested at 20 h.p.i., subjected to Western blot analysis and quantitative real time RT-PCR. Mock infection without GSK treatment was set as control. The intensities of phospho-eIF2α and CHOP band were normalized to eIF2α or β-actin, respectively, and shown as fold change of GSK (+:-).

**Figure S4. AKT protects cells from apoptosis and JNK promotes cell apoptosis.** (A-D) Involvement of AKT JNK, p38, or ERK1/2 in NDV-induced apoptosis. HeLa cells were infected NDV, followed by treatment with DMSO, 10µM AKT inhibitor LY294002, 7.5µM JNK inhibitor SP600125, 10µM p38 inhibitor SB203580, or 10µM ERK1/2 inhibitor U0126. Mock infection was included as control. Cell lysates harvested at 20 h.p.i.. and analyzed with Western blotting by using indicated antibodies. The intensities of CASP3-C, PARP-C, or NP were normalized to β-actin, and shown as fold change of inhibitor (+:-). The protein bands intensities of NDV-infected cells with DMSO treatment were set as 1.

**Figure S5: IRE1α regulates NDV proliferation and expression of ER chaperones/ERAD components.** HeLa cells were transfected with sic or siIRE1α for 48 h (A), or transfected with plasmid pCMV or pCMV-IRE1α for 24 h (B), followed with NDV infection. The cells were harvested at 20 h.p.i. and analyzed with quantitative real time RT-PCR, to detect ERdj4, p58^IPK^, and EDEM1 mRNA (A-B, left and right column panels). mRNA and virus titer data represent means ± SD of three independent determinations. **p*<0.05, ***p*<0.01; ****p*<0.001.

**Figure S6: Inhibition of JNK suppresses the expression of cytokines.** HeLa cells were infected with NDV, followed by treatment with DMSO or 7.5 µM JNK inhibitor SP600125, and harvested at 20 h.p.i. (A). In parallel, HeLa cells were transfected with siJNK or sic for 48 h, followed by NDV infection for 20 h (B). Mock infection was included as control. The levels of IFN-β, TNF-α, IL6, and IL8 mRNA were determined with quantitative real time RT-PCR. mRNA data represent means ± SD of three independent determinations. **p*<0.05, ***p*<0.01; ****p*<0.001.

**Figure S7. Working model of UPR associated apoptosis and inflammation during NDV infection.** NDV replication activates PKR and three UPR branches. PKR and PERK phosphorylates eIF2α, induces the expression of CHOP. CHOP promotes apoptosis via reducing the level of anti-apoptotic protein BCL-2 and MCL-1，stimulating JNK signaling, and restricting the pro-survival AKT signaling. Meanwhile, NDV infection activates IRE1α, which promotes apoptosis via XBP1 splicing and JNK signaling cascade. JNK also contributes to cytokines secretion. XBP1 splicing and ATF6 cleavage promotes the expression of chaperones and ERAD components, thereby alleviating the ER stress. Overall, UPR triggers apoptosis/inflammation and promotes NDV release.
