# Supplementary figures and images for: eIF2α-CHOP-BCl-2/JNK and IRE1α-XBP1/JNK signaling promote apoptosis and inflammation and support the proliferation of Newcastle disease virus
Source: Cell Death Dis. 2019 Nov 26;10(12):891. doi: 10.1038/s41419-019-2128-6 (PMC6877643; doi:10.1038/s41419-019-2128-6)

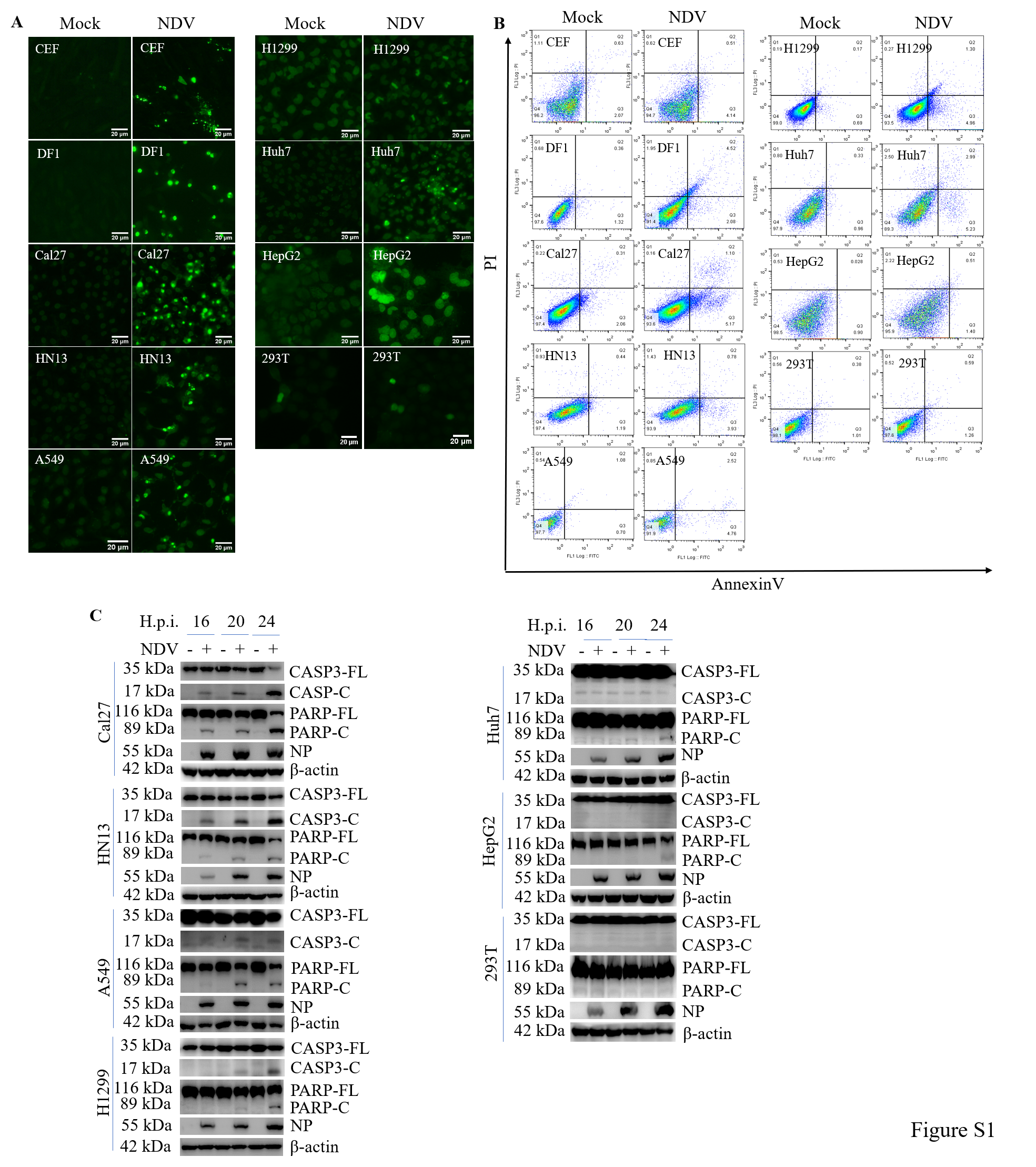

Supplement: Supplementary file 2 — Figure S1 [file 41419_2019_2128_MOESM2_ESM.png]

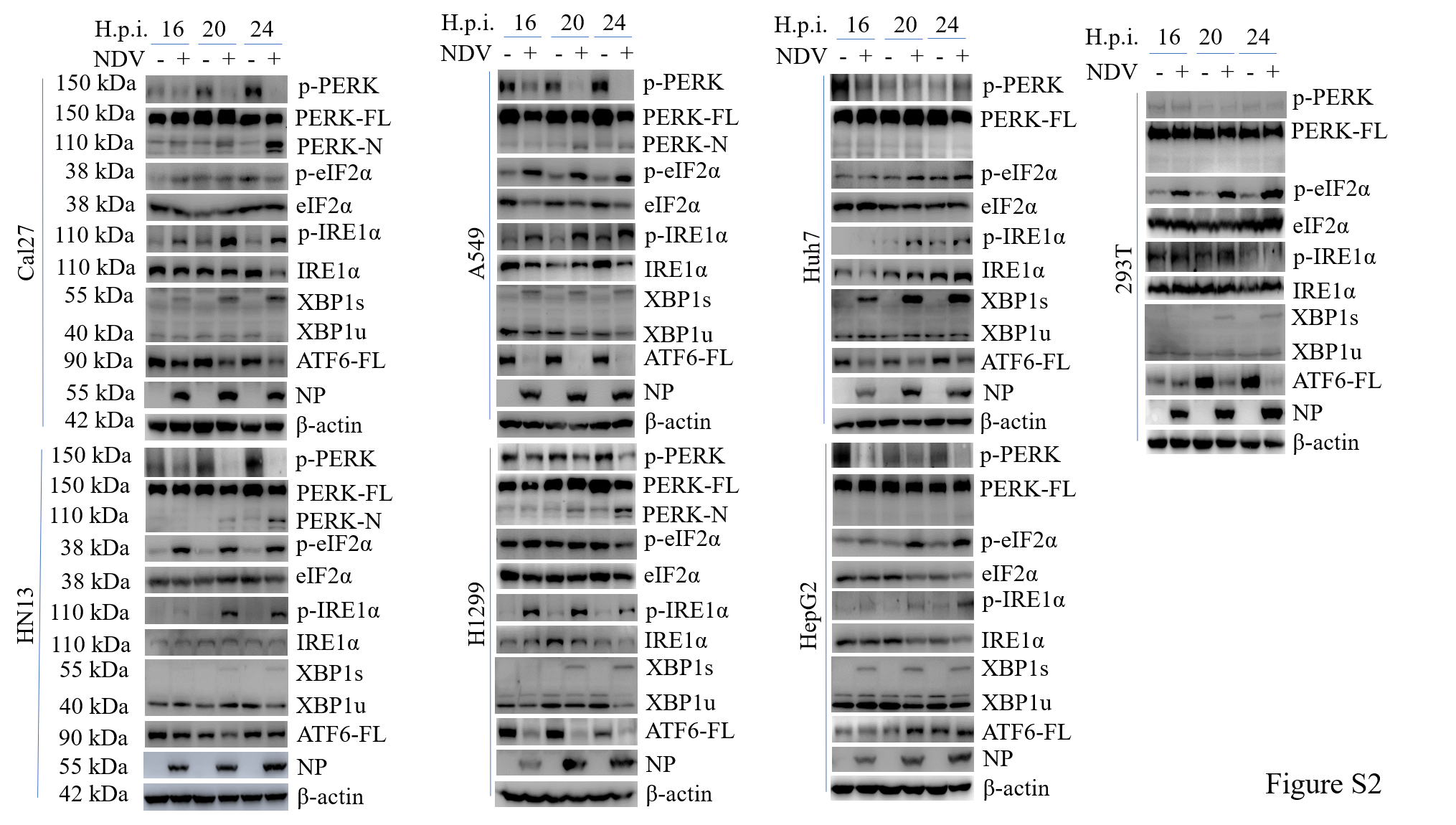

Supplement: Supplementary file 3 — Figure S2 [file 41419_2019_2128_MOESM3_ESM.png]

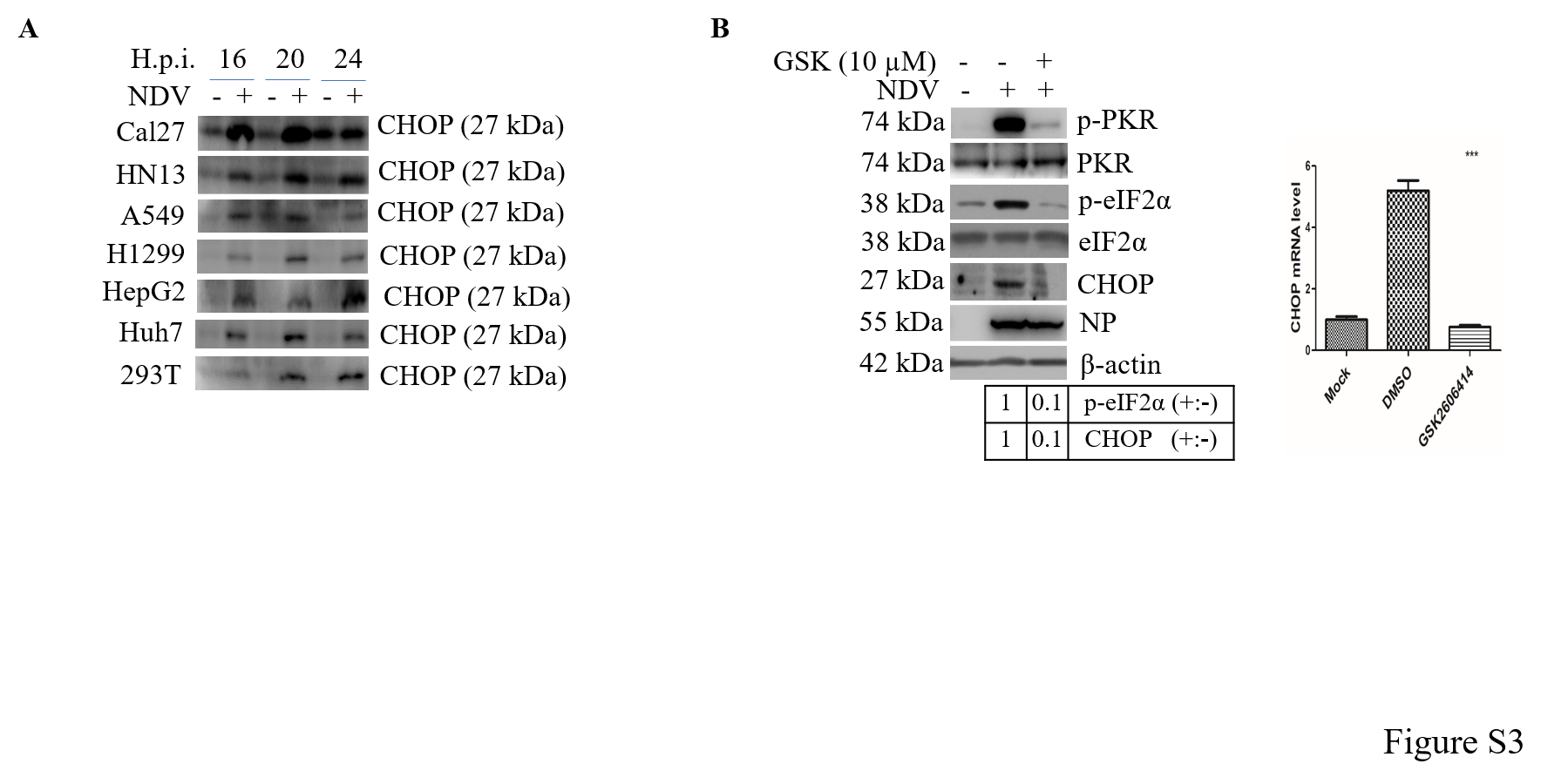

Supplement: Supplementary file 4 — Figure S3 [file 41419_2019_2128_MOESM4_ESM.png]

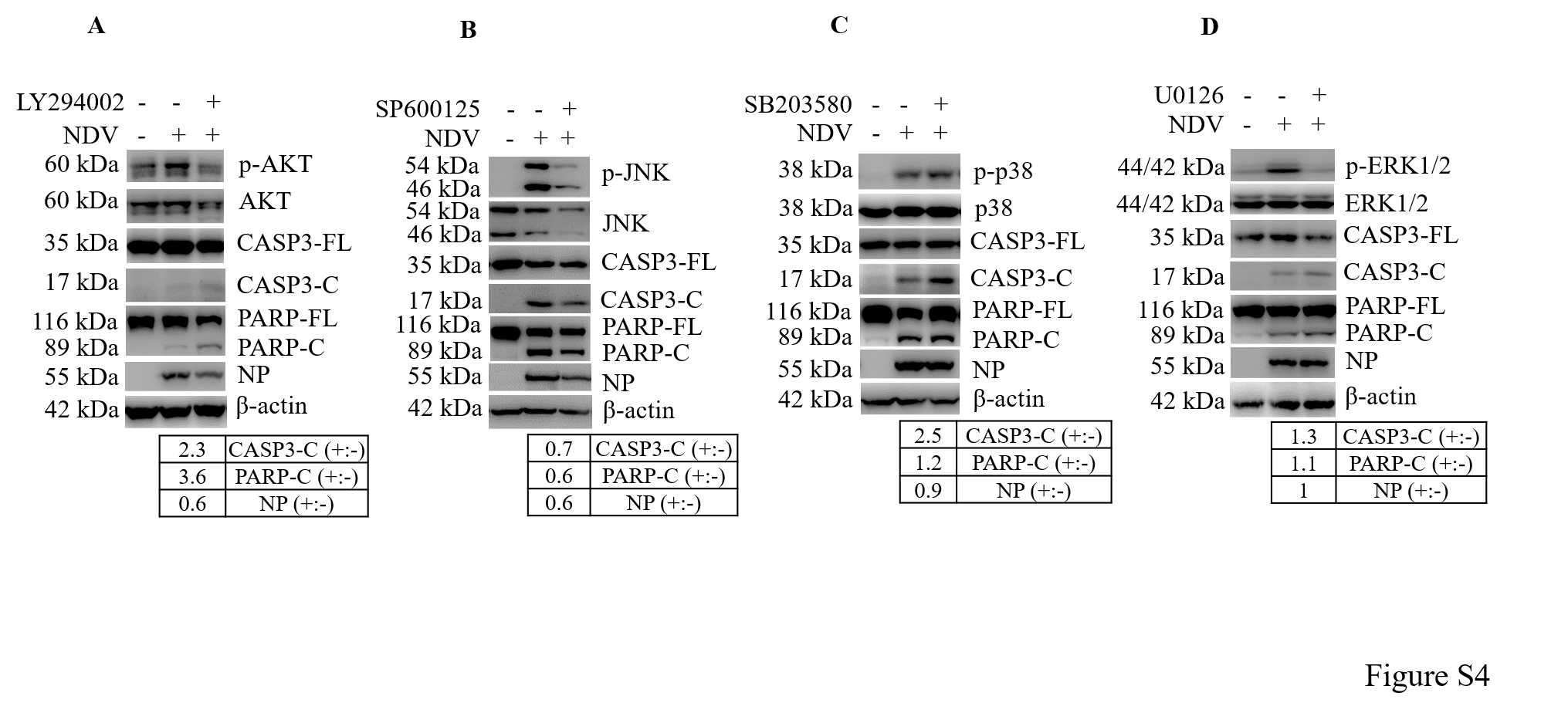

Supplement: Supplementary file 5 — Figure S4 [file 41419_2019_2128_MOESM5_ESM.png]

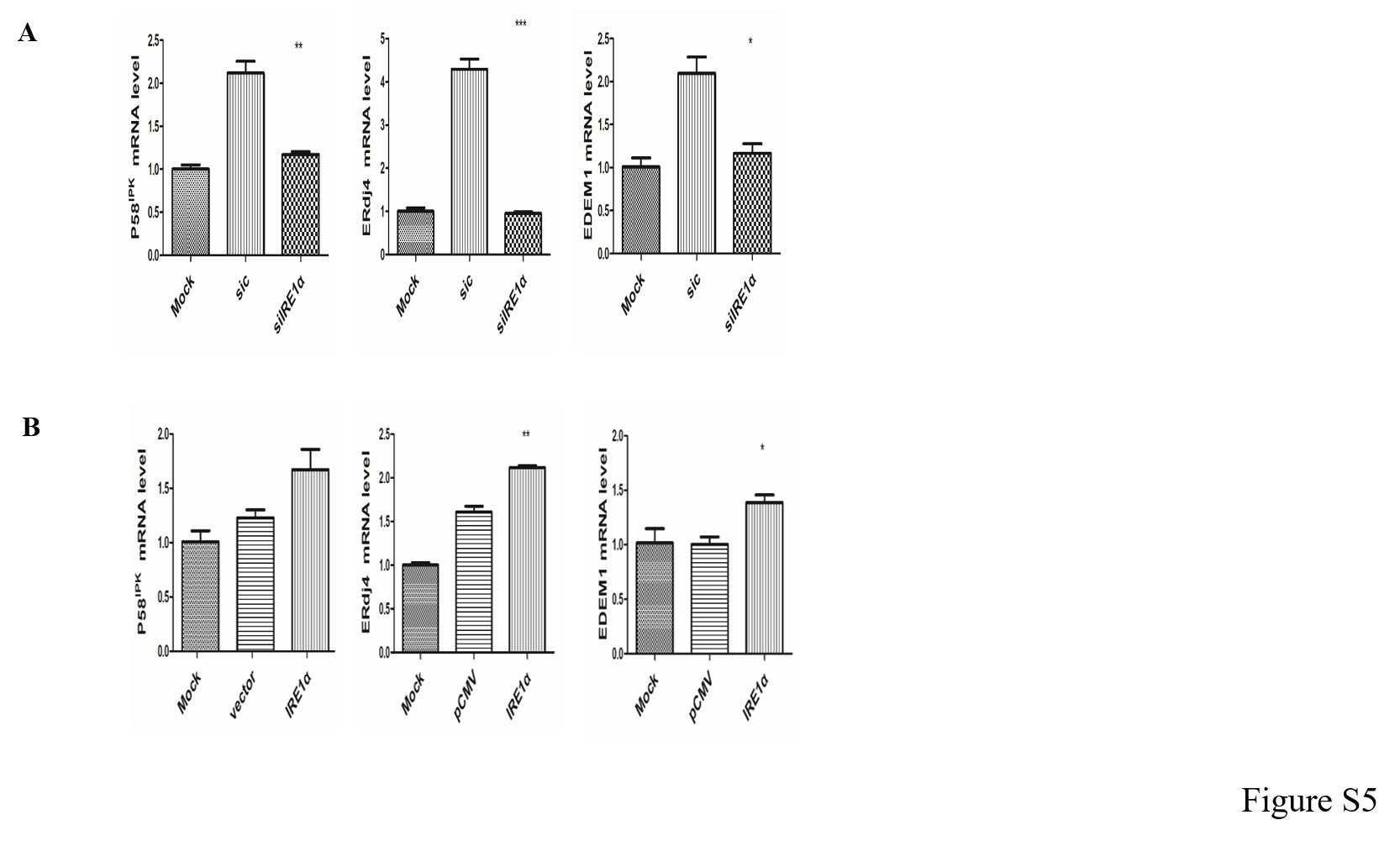

Supplement: Supplementary file 6 — Figure S5 [file 41419_2019_2128_MOESM6_ESM.png]

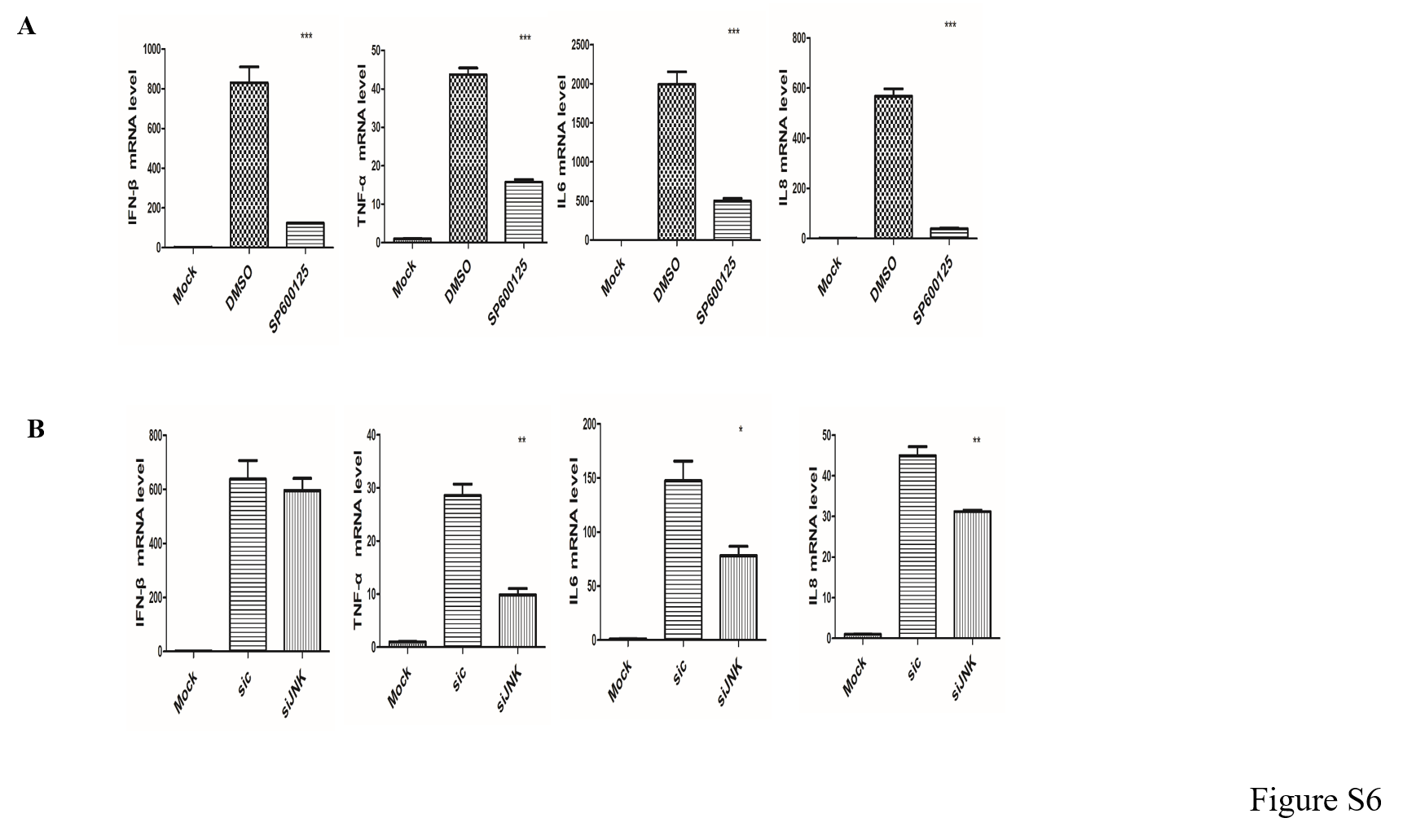

Supplement: Supplementary file 7 — Figure S6 [file 41419_2019_2128_MOESM7_ESM.png]

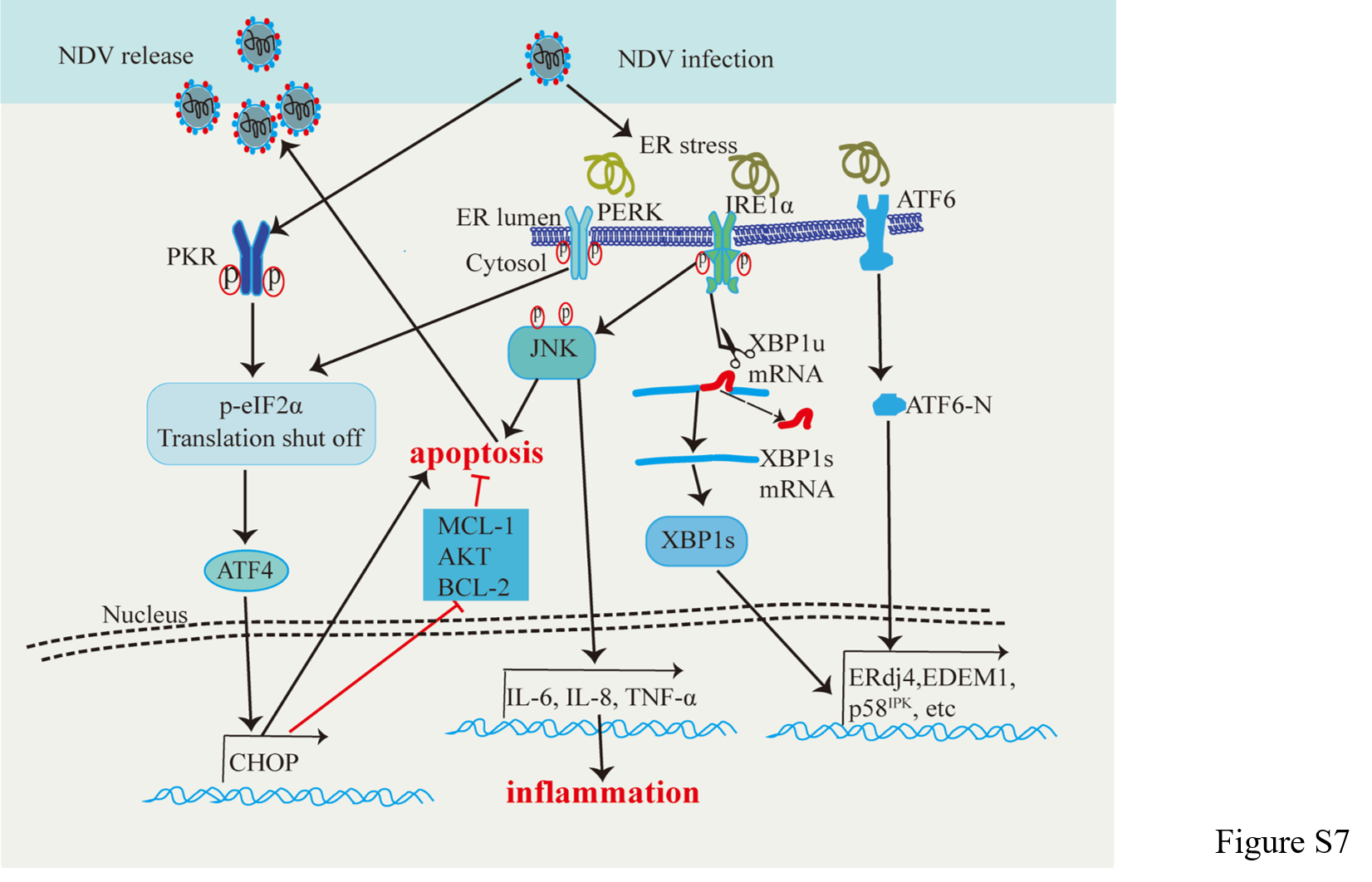

Supplement: Supplementary file 8 — Figure S7 [file 41419_2019_2128_MOESM8_ESM.png]
